# Spectrofluorimetric determination of tapinarof via Zn (II) complexation and assessment of its topical dosage application

**DOI:** 10.1186/s13065-024-01271-7

**Published:** 2024-09-10

**Authors:** Hesham Salem, Mahmoud A. Abdelmajed, Hoda Madian, Nadeen Emad, Sara Osama, Amir Ata, Ebtihal Samir

**Affiliations:** Pharmaceutical Chemistry Department, Faculty of Pharmacy, Deraya University, New Minia, Egypt

**Keywords:** Tapinarof, Zinc, Sodium dodecyl sulfate, Metal complexation, Ointment, Green analytical process Index

## Abstract

Topical tapinarof is used to treat plaque psoriasis (a skin disease in which red and scaly patches form are appeared on some areas of the body). The goal of the current research is to establish a facile and rapid fluorimetric technique for tapinarof analysis. The approach relied on the reaction between the drug and zinc ion through metal complexation to produce a highly-fluorescent product. The fluorescence was further enhanced by adding sodium dodecyl sulfate, and it was observed at 542 nm following excitation at 497 nm. With a correlation coefficient of 0.9997, the association between emission intensity and tapinarof concentration was linear between 2.0 and 120 ng mL^−1^. 1.021 ng mL^−1^ was the quantitation limit while 0.366 ng mL^−1^ was the detection limit. The buffer type, pH and concentration, type of surfactant and concentration, and finally the diluting solvent were among the reaction conditions that were closely examined and it was found that the optimum conditions were obtained upon employing teorell-stenhagen buffer optimized at pH 6.0, 1.38 × 10^–2^ M SDS and distilled water as a solvent are the suitable choice. With great precision and reliability, the drug under study was quantified using this method in ointment formulations. The proposed method's level of greenness was assessed using two methodologies: the analytical greenness metric (AGREE) and the Green Analytical Procedure Index (GAPI), with good recovery results ensuring high efficiency of the proposed approach on analysis of ointment without any interference from additives and excipients.

## Introduction

While topical medications remain the cornerstone of care for those with mild-to-moderate psoriasis, there haven’t been many advancements in this field over the past 20 years. Despite having several drawbacks, corticosteroids and vitamin D analogues continue to be the most often prescribed medications. In May 2022, the U.S. Food and Drug Administration (FDA) authorized Tapinarof (TAP), also known as benvitimoid, for the treatment of people (18 years of age and older) with plaque psoriasis, which is the most prevalent chronic immune-mediated subtype [1–3]. Unlike steroid-based treatments, TAP is a nonsteroidal topical drug that belongs to a unique class of naturally occurring aryl hydrocarbon receptor (AHR) agonists, making it the first agent of its kind.

**Fig. 1:**
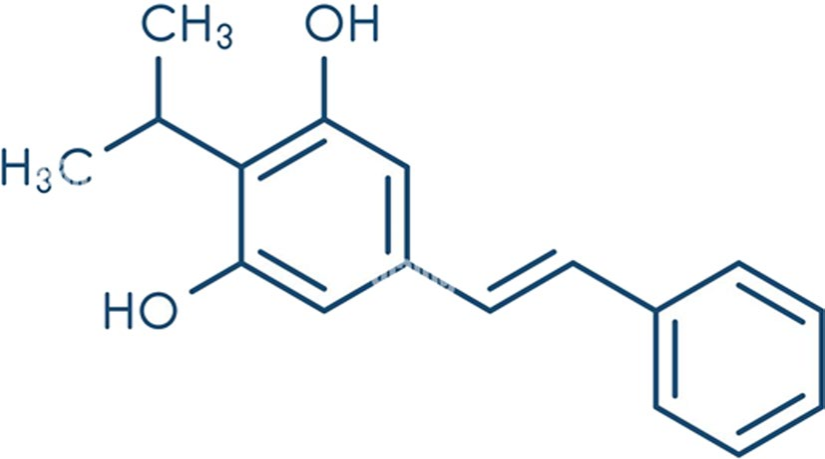
Chemical structure of Tapinarof

Chemically, it is a 3, 5-Dihydroxy-4-isopropylstilbene, as illustrated in (Fig. 1). There was just one HPLC paper that was documented in regard to reported analytical publications for determination of the stated medication in its therapeutic dosage forms. Related to the reported chromatographic technique, a time-consuming, tedious preparation process and large volume of waste organic solvent produced during the analytical procedure should be undergoing [4].

However, spectrofluorimetric approaches represent a very simple and inexpensive technology for drug analysis that may be environmentally benign if the reagents and solvent are chosen appropriately [5–13], there was insufficient work published spectrofluorimetric approach for TAP estimation. By metal complexing with Zn ions to sensitize the drug's native fluorescence and then encapsulating it in a micelle, the novel spectrofluorimetric approach for TAP quantification was established and achieved excellent selectivity and sensitivity. If metal binding takes place on the analyte's light-emitting moiety, the metal complex creation of the analyte may enhance its fluorescence activity [14–18]. Increasing the analyte's molecular rigidity and reducing its free rotation will increase its fluorescence [19], improving its micro-environment or protecting its excited state from the non-radiaive deactivation processes [20].

So the current work aimed to establish the first spectrofluorimetric method used for quantification of TAP by formation a complex with Zn ions and the fluorescence was enhanced in the presence of sodium dodecyl sulphate (SDS) micelle system, that through a facile and rapid methodology which has good selectivity and sensitivity.

## Experimental

### Apparatus

The SCINCO FluoroMate (FS-2, Korea) was used to measure all fluorimetric emission values during the current technique. The quartz cell’s path length was one centimeter, and the light source was a 150 W Xenon-arc lamp. For both the excitation and emission, the slit width was 5 nm. To assess pH, an Adwa (AD 11, Romania) pH meter was employed and a 4000 rpm (Bramsen ECCO, Germany) centrifuge was also utilized.

### Chemicals, reagents and dosage forms

Analytical or pharmaceutical grade reagents and substances were utilized. TAP with a purity of 101.34% [4]. VTAMA^®^ cream (Dermavant Sciences, U.S.A), labelled to contain 1% TAP per 60 g cream. El-Nasr for Chemicals and Pharmaceuticals Company (Abo Zabaal, Cairo, Egypt) supplied sodium dodecyl sulfate (SDS), hydrochloric, citric, and phosphoric acids, sodium hydroxide, as well as the salts of chloride of zinc, magnesium, calcium, cobalt, copper, and barium. A freshly made 0.53 M Zn^2+^ solution (or 0.025% g w/v) was made via dissolving 25 mg of the reagent within 100 ml of distilled water. Via dissolving 5 g within 100 mL of distilled water, an aqueous solution of 0.17 M of (5% g w/v) of SDS was created.

Teorell-Stenhagen buffer with pH values between (2.0–9.0) were prepared by adding sodium hydroxide, phosphoric acid, and citric acid in the appropriate amounts to 0.1 M solutions. The pH was adjusted to the necessary value using a 0.1 M solution of hydrochloric acid.

### Preparation of standard drug solutions

In a 100 mL volumetric flask, 10.0 mg of the authentic was mixed with distilled water to prepare a stock solution of 100.0 µg mL^−1^ of TAP. Further dilutions of the used stock were made using the same diluent to output the experimental working solutions. When kept in the refrigerator, the produced solutions were shown to obtain stability for at least seven days.

### Procedures

#### Procedure for general analysis

**Fig. 2:**
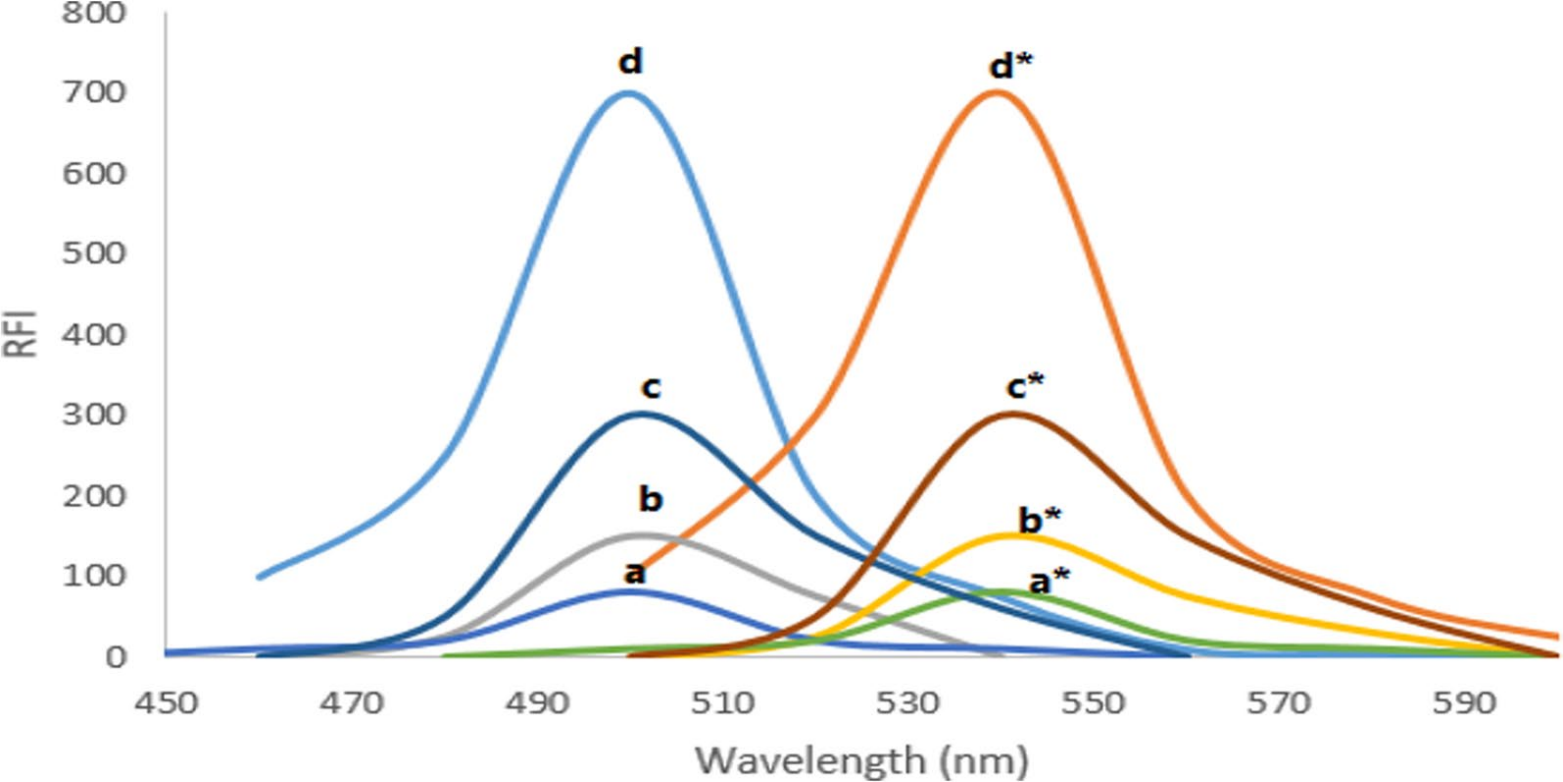
Excitation and emission spectra of (**a**, a*) Zn (II)-SDS reagent, (**b**, b*) 100 ng mL^−1^ TAP in water, (**c**, c*) TAP-Zn (II) and (**d**, d*) TAP-Zn (II)-SDS system. *RFI means relative fluorescence intensity

Appropriate amounts of the TAP standard solution, providing a final concentration within the range of 2–120 ng mL^−1^, were placed in a series of volumetric flasks with a 10 mL capacity. After adding 1.0 mL of 0.53 Zn (II) solution and two milliliters of Teorell-Stenhagen buffer (pH 6.0), one milliliters of 0.17 M SDS was added. After adding water to fill the flasks to the exact line, the mixture was carefully allowed to settle for 10 min at room temperature. Without the addition of the cited drug, similar procedures were carried out to prepare the blank experiment. Following excitation at 497 nm, the fluorescence intensity were measured at 542 nm (Fig. 2). The calibration graph was built via plotting the relative fluorescence intensity against the drug concentration in the final solutions.

#### Job’s method for estimation of the stoichiometric ratio

**Fig. 3:**
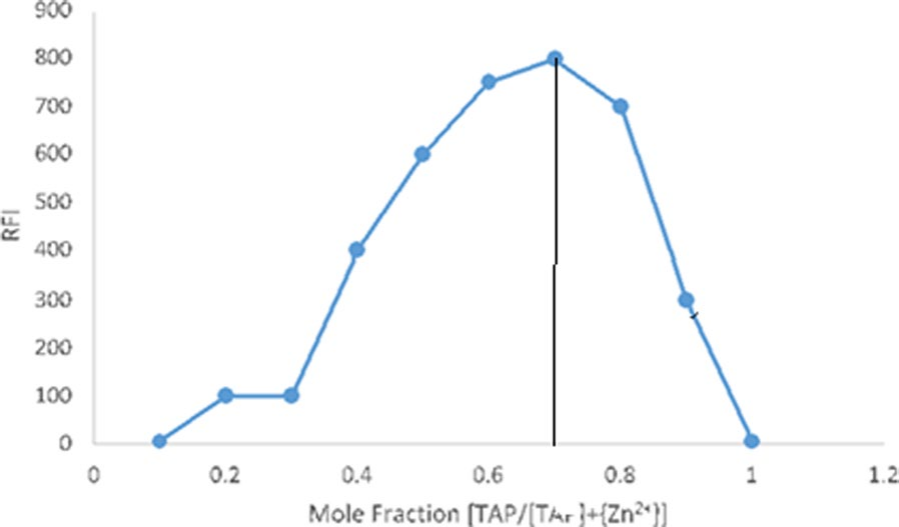
Job’s method for estimation of the stoichiometric ratio

Before general procedure was proceed, equimolar concentrations of Zn (II) and TAP were prepared (5.3 × 10^−4^ M), various complementary quantities of the pair solutions were used and the total volume in all situations should be 1.0 mL. Finally, fluorescence values against the mole fractions of TAP allowed Job's plot to be constructed. As shown in (Fig. 3).

#### Analysis of topical dosage form

A five-gram of the cream was transferred to a 50-mL volumetric flask, then 35 mL of methanol was added and heated to 60 °C with constant shaking for complete melting and extraction of the studied active constituent without any additives or excipients. Then, cooling to room temperature was employed and completed to mark with Methanol. The solution produced a translucent supernatant solution after centrifugation for 10 min at 5000 rpm. A portion of the supernatant was diluted with methanol to produce the final concentrations, and general analytical procedures was allowed.

## Results and discussion

### Approach fluorescence spectrum

Figure 2 displays the fluorescence spectrum (curves b and b*) of 100 ng mL^−1^ TAP in aqueous solution. The medication exhibits fluorescence intensity at an emission wavelength of 542 nm and an excitation wavelength of 497 nm. Curves c and *c show how the complex formation with Zn (II) ions improved the drug's excitation and emission spectra. Curves d and d* make it evident that the combination of Zn (II) and SDS significantly increased the fluorescence of TAP by a factor of 4.0 and about seven folds of the native fluorescence of TAP. Curves a and a* fortunately show that neither the metal ion nor the solutions of surfactant showed any significant fluorescence. Thus, in the current work, Zn (II) and SDS were both utilized as enhancers for the fluorescence of TAP. The final solution in each of these tests included 0.02 M Teorell-Stenhagen buffer of (pH 6.0), 1.38 × 10–2 M SDS, and 0.053 M Zn (II).

### Optimization of the developed method

All the experimental parameters were optimized and summarized in Table 1, including influence of diluting solvents, the type of buffer solution and the type of surfactants,

**Table 1:** The influence of diluting solvents, the type of buffer solution (pH 6.0), and the type of surfactants (0.2 M solution) on fluorescence intensity of the complex of 60 ng mL^−1^ TAP with 0.53 mM Zn (II)

Solvent	RFI	Buffer type	RFI	Surfactant	RFI
Water	701	Teorell–Stenhagen	723	SDS	697
Propan-2-ol	280	Britton Robinson	364	β-CD	332
Methanol	315	Borate	90	CTMAC	284
Ethanol	210	Acetate	199	CMC	175
Dioxan	138	Phosphate	189	PEG 6000	286
DMSO	112			None	111

#### Buffer optimization

**Fig. 4:**
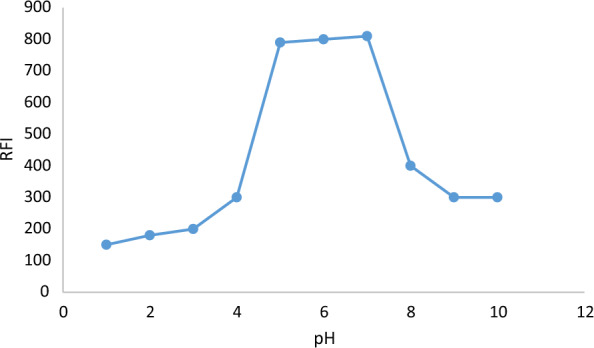
The influence of pH on the fluorescence intensity of reaction products of TAP (80 ng mL 1) with 5.5 10 4 M Zn^2+^ and 0.15 M SDS

A thorough analysis was conducted on the impact of several buffer solutions, such as Teorell-Stenhagen, acetate, Britton-Robinson, borate, and phosphate buffers, on the fluorescence. Teorell-Stenhagen proved to be the most suitable buffer system, yielding the highest values of fluorescence, as shown in Table 1. Using Teorell-Stenhagen buffer solutions with varying pH values (2.0–9.0), pH effect on the emission of the produced metal chelate was further evaluated [21]. At pH 5.0–7.0, the maximum fluorescence value was reached, outside of this range the solution's fluorescence gradually decreased leading to the designation of pH 6.0 as the optimal pH, as shown in (Fig. 4). While maintaining the pH of all solutions at 6.0, the effects of varying the used buffer's volume were examined using varying volumes (0.4–4.0 mL). At 1.5–2.5 mL, the observed emission intensities reached their maximum values. Lower fluorescence intensity was observed with larger quantities of the buffer solution; this could be because the solution's salt strength increased. Consequently, 2 mL of buffer at pH = 6 was chosen to be the optimum buffer condition.

#### Optimization of the chelating agent

With the use of 0.5 M aqueous solutions of various metal ions, the complexation of TAP was investigated. The maximum fluorescence intensity was recorded by Zn (II) at 542 nm, whereas the values for the other metal ions were Ba (II), Cu(II), Co(II), Ca(II), and Mg(II) correspondingly, 310, 189, 298, 301, and 298. Next, under investigation of metal concentration, different volumes of 0.53 M Zn (II) were employed resulting that increasing the zinc ion volume increased the fluorescence intensity correspondingly with values peaking at 1.0 mL. The fluorescence did not significantly change at higher metal volumes. Consequently, 1.0 mL was the optimum volume of the 0.53 M zinc ion solution.

#### Optimization of the surfactant type

It is well known that adding surfactants at concentrations greater than the critical micelle concentration enhances fluorophores’ capacity to produce light. Thus, different types of surfactants were added to the reaction solution in order to test them. The investigation included both anionic (sodium dodecyl sulfate, SDS) and cationic (cetyltrimethyl ammonium chloride) surfactants, along with non-ionic polymers (polyethylene glycol 6000) and macromolecules such carboxymethyl cellulose and β-cyclodextrin. The TAP-Zn (II) complex’s emission intensity increased significantly in response to all of the chemicals that were investigated, but in this experiment, SDS was chosen as a fluorescence enhancer since it had the greatest effect [22–27].

#### Optimization of SDS volume

**Fig. 5:**
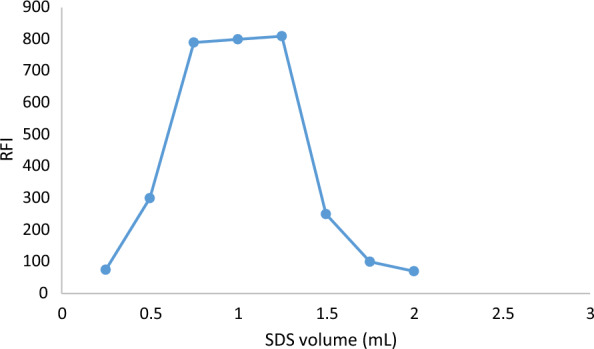
Effect of volume of 0.15 MSDS reagent on the fluorescence intensity of the reaction product of TAP (80 ng mL 1)

A key role for the surfactant is played in the process of enhancing TAP's natural fluorescence. As a result, different quantities of 0.17 M SDS within the range of 0.2–2.0 ml were included in the investigation. Increasing the volume of surfactant (0.17 M SDS) caused a discernible increase in emission intensity, and utilizing 0.5–2.0 mL produced the maximum fluorescence. Therefore, 1.0 mL of surfactant was selected, resulting in a final concentration of 1.38 × 10–2 M SDS, all data was provided in **(**Fig. 5**).**

#### Solvent optimization

Many solvents were tested in order to determine which one was the best for dissolving the produced TAP complex with Zn^2+^ in the system of SDS micelle. Solvents such as dimethyl sulfoxide (DMSO), ethanol, 2-propanol, distilled water, methanol, and dioxan were checked in the experiment. DMSO and dioxan both produced low emission levels. These solvents raise both the micelle dissociation degree and the critical micelle concentration [28]. When compared to the intensities achieved with distilled water, the values obtained with ethanol, methanol, or 2-propanol remained unsatisfied, despite their relative high intensities. These alcohols' detrimental effects on micelle aggregation may be the cause of the low fluorescence intensity values observed when they are used. Distilled water was therefore selected as a solvent to dilute the chelate that was generated since it produced the highest emission intensity. It is important to note that distilled water is the most affordable and ecologically friendly solvent available, making the suggested technique both cost-effective and compliant with green chemistry guidelines.

At various intervals, the fluorescence of the generated complex was observed. Since there was no discernible change in the fluorescence intensity for at least three hours, the complex formed quickly and was very stable.

### Job’s method employment

Using Job's technique [29], the stoichiometric ratio between TAP and Zn (II) was investigated. Equimolar concentrations of TAP and Zn (II) (5.3 × 10^–4^ M) were used for the overall process. Given that the maximum fluorescence was observed at (0.666) mole fraction, a ratio 1:2 of the combination between Zn (II) ions and TAP was deduced as illustrated in (Fig. 3).

### Mechanism of the studied reaction

**Fig. 6:**
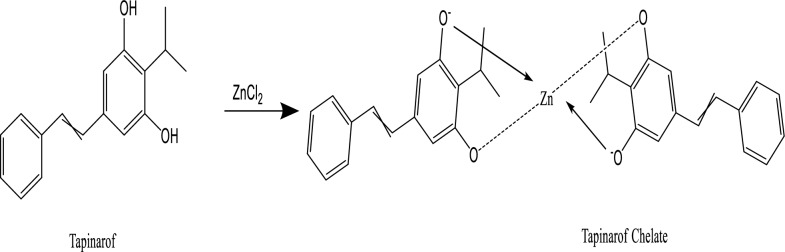
The structure of the chelate formed between TAP and ZN (II) ion

According to the complex that forms between Zn (II) ions and the two oxygen atoms in TAP, the natural emission activity of the aqueous solution of TAP could be considerably enhanced by the addition of Zn (II) solution, as shown in Fig. 6. As a result, the fluorescence activity increased along with the molecular stiffness, furthermore, the fluorophore's incorporation into the micelle's core improved its fluorescence in two different ways. The first may arise from strengthening the micelle's internal environment by raising its viscosity, whereas the second may be related to protecting the fluorophore's excited singlet state from the solvent's quenching action and preventing the non-radiative deactivation process. As a result, the surfactant (SDS) and Zn (II) ions both produced a synergistic increase in TAP's fluorescence.

### Approach validation

Based on ICH [30] guidelines, the proposed approach was fully validated. The parameters under investigation are listed below.

#### Linearity, range and Limits of detection and quantitation

Various TAP concentration levels were analyzed using the general analysis approach and comparing with the produced fluorescence intensity resulting a calibration curve. With a correlation value (r) of 0.9997, the relationship’s remarkable linearity was demonstrated within the drug concentration range of 2–120 ng mL^–1^. Table 2 displays the outcomes of the estimation of the analytical parameters.

**Table 2:** Analytical parameters for the determination of the TAP drug with the proposed method

Parameter	Value
Excitation wavelength, λex (nm)	497
Emission wavelength, λem (nm)	542
Linear range (ng mL^−1^)	2–120
Slope	197.31
Intercept	4.917
Coefficient of determination, r^2^	0.9997
Limit of quantitation, LOQ (ng mL^−1^)	1.021
Limit of detection, LOD (ng mL^−1^)	0.366

To evaluate the sensitivity of the approach, both quantitation (LOQ) and detection (LOD) limits were calculated. This formula was used for the estimation: L = n SD/b where n is 10 for LOQ and 3.3 for LOD, L is the limit, b is the slope of the calibration line, and SD is the standard deviation of the intercept. The obtained values of LOD and LOQ were 0.366 and 1.021 ng mL^−1^, respectively, indicating that the suggested approach has excellent sensitivity. (LOD) and (LOQ) are used to describe the smallest concentration of a measure and that can be reliably measured by an analytical procedure. LOD is determined by utilizing test replicates of a sample known to contain a low concentration of analyte. The LOQ may be equivalent to the LOD or it could be at a much higher concentration.

#### Accuracy, precision and robustness of the method

To assess the accuracy along with precision of the suggested approach, the overall procedure was applied to standard TAP solutions with various concentrations. For the intra-day and inter-day levels, the analysis was done three times: once on the same day and once over the course of three days. For every example, the percentage recoveries (%R) and the relative standard deviations (%RSD) were computed. The data in Table 3 showed that the recommended method's repeatability was satisfactory.

**Table 3:** Evaluation of accuracy, intraday, and inter-day precisions of the analytical procedure of the determination of TAP

Analysis	Taken (ng mL^−1^)	% Recovery ± RSD
Accuracy	20	100.42 ± 1.06
	40	99.49 ± 0.943
	60	100.43 ± 1.21
Intraday precision	5	100.67 ± 0.89
	10	98.79 ± 1.11
	15	99.67 ± 0.85
Interday precision	90	99.48 ± 1.00
	100	99.40 ± 1.39
	110	100.03 ± 0.97

The impact of making a little modification to the overall procedure's parameters was investigated. The variables under test were the pH of the buffer, the volume of 0.53 M Zn(II), and the surfactant content (0.17 M SDS). The obtained (%R) and SD values showed that there was no discernible change in either of the studied variables in the analytical outcomes, confirming the robustness of the described approach, as shown in Table 4.

**Table 4:** Robustness of the proposed method for determination of TAP (100 ng mL^−1^)

Parameters	% Recovery ± SD
pH of buffer	
6.2	100.10 ± 00.65
6.0	100.86 ± 1.75
5.8	98.96 ± 1.64
Zn^2+^ volume	
0.8	99.50 ± 0.75
1.0	98.69 ± 1.68
1.2	99.43 ± 0.85
SDS volume	
0.8	101.70 ± 1.75
1.0	99.68 ± 0.43
1.2	100.93 ± 1.75

### Developed approach application

#### Quantification of VTAMA^©^ cream

The VTAMA^®^ cream was successfully quantified using the established procedure with highest % R values and simultaneously it was analyzed using a published HPLC technique. The procedure included a column temperature of 30 °C, a mobile phase of 90% methanol, and a flow rate of 1.0 mL min^−1^. It was shown that 2.88 min was the retention time for TAP, while the concentration range is 5 to 30 µg mL^−1^ [4]. By statistically comparing the methods' findings and estimating the Student's t-test and F-test values at the 95% confidence level, the suggested method's accuracy and precision were assessed (Table 5). The predicted values of the t- and F-values were not more than the tabulated ones, indicating that the suggested procedure was accurate and exact, according to the results. Furthermore, these findings demonstrated that the excipients included in these formulations had no effect on the recommended method’s analytical performance.

**Table 5:** Application of the proposed method for the determination of the TAP in dosage form

% Recovery ± SD^a^		t-test^b^	F-value ^b^
Proposed method	Reported method^C^		
99.78 ± 0.98	100.04 ± 0.94	1.774	1.029

^a^The value is the average of three determinations and SD is the standard deviation

^b^Theoretical value at 95% confidence limit; F = 19.00 and t = 2.78

^c^Chavva and Gosu [4], reported analytical article for determination of the cited drug in its pharmaceutical dosage forms by HPLC

### Green features of suggested method

**Fig. 7:**
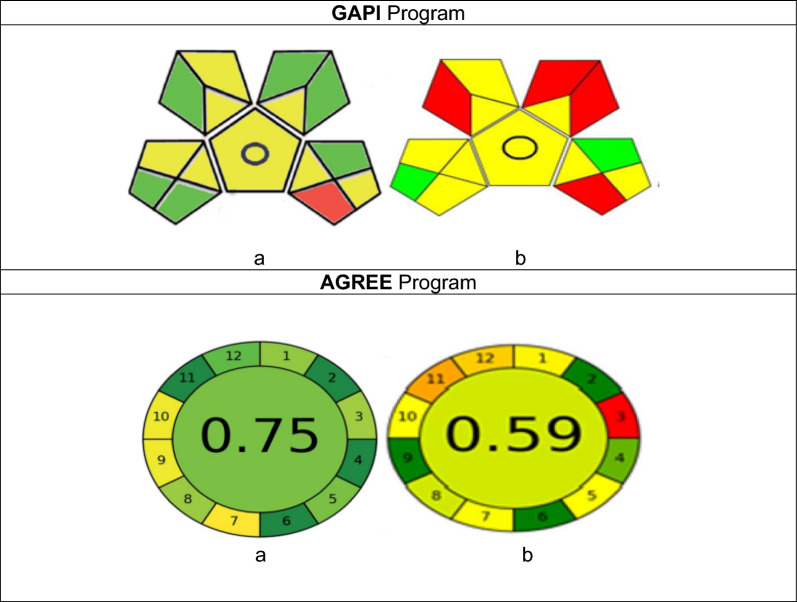
GAPI and AGREE pictograms for a the proposed spectrofluorimetric method and reported HPLC method**, a** proposed method, **b** for Reference method

Many tools are now available to evaluate and contrast how green different analytical procedures are. The new green analytical process index (GAPI) [31, 32] has the unique advantage of covering the entire analytical method as opposed to the previous analytical eco-scale [33]. It consists of five pentagrams, each of which denotes a step in the analytical technique: sample preparation and collection; chemicals and solvents; applied equipment; and the goal of the analytical technique. Three color codes are used by GAPI: red denotes a significant environmental threat, while yellow and green denote a lower risk and increased greenness. The proposed spectrofluorimetric method was compared with HPLC published technique that already existed. Figure 7 shows that the recommended method in its GAPI had seven green and eight yellow pentagrams. The GAPI pentagrams state that the recommended spectrofluorometric method is an environmentally benign and green alternative to the chromatographic procedure that has been previously described. The most troublesome phase for most analysts, the extraction step, was eliminated from the prior technique. Green micellar solvents were also used, along with instrumentation that required less energy and produced less waste.

## Conclusion

The cited drug, TAP, could be quantified in its authentic and topical dosage form via a facile, rapid and cost-effective spectrofluorimetric approach. The developed approach doesn't require any previous extraction or consuming of large volumes of costly solvents prior to estimation. Using distilled water as a significant green diluting solvent allows for environmental safety. In many quality-control laboratories, it could be easily used in the routine analysis of the medication under examination owing to its affordability and environmental friendliness. Finally, AGREE and GAPI pentagrams state that the recommended spectrofluorimetric method is an environmentally benign and green alternative technique to the chromatographic procedure.

## Data Availability

No datasets were generated or analysed during the current study.

## Abbreviations

AHR: Aryl Hydrocarbon Receptor
DMSO: Dimethyl Sulfoxide
FDA: Food and Drug Administration
GAPI: Green Analytical Process Index
ICH: International Conference of Harmonization
LOD: Limit of Detection
LOQ: Limit of Quantitation
SDS: Sodium Dodecyl Sulfate
TAP: Tapinarof
